# Differences in Subjective Memory Impairment, Depressive Symptoms, Sleep, and Physical Activity in African American and Asian American Elderly

**DOI:** 10.3390/brainsci11091155

**Published:** 2021-08-31

**Authors:** Minsun Lee, Jin-Hyeok Nam, Elizabeth Yi, Aisha Bhimla, Julie Nelson, Grace X. Ma

**Affiliations:** 1 Center for Asian Health, Lewis Katz School of Medicine, Temple University, Philadelphia, PA 19140, USA; minsun.lee@temple.edu (M.L.); jnam@temple.edu (J.-H.N.); elizabeth.yi@temple.edu (E.Y.); aisha.bhimla@temple.edu (A.B.); 2 Philadelphia Senior Center, Philadelphia, PA 19147, USA; jnelson@philaseniorcenter.org; 3 Department of Clinical Sciences, Lewis Katz School of Medicine, Temple University, Philadelphia, PA 19140, USA

**Keywords:** subjective cognitive impairment, Asian American, African American, ethnic difference

## Abstract

**Background**: Subjective memory impairment (SMI) is associated with negative health outcomes including mild cognitive impairment and Alzheimer’s disease. However, ethnic differences in SMI and disparities in risk factors associated with SMI among minority populations are understudied. The study examined the ethnic differences in SMI, whether SMI was associated with depressive symptoms, sleep, and physical activity (PA), and whether the associations vary across racial/ethnic groups. **Methods**: Participants included 243 African and Asian Americans (including Chinese, Vietnamese, and Korean Americans) aged 50 or older. Demographic information, SMI, depressive symptoms, daily sleeping hours, and PA levels were assessed. **Results**: Vietnamese Americans reported the highest SMI score. Depressive symptoms, sleeping hours, and PA levels were significantly associated with SMI. Depressive symptoms were the only significant factor across all ethnic groups. Significant interaction effects were found between ethnicity and health behaviors in predicting SMI. In particular, Vietnamese American participants with greater depressive symptoms and physical inactivity were significantly more likely to experience SMI compared to other ethnic groups **Conclusions**: Our findings demonstrate ethnic differences in SMI and its association with depressive symptoms, sleep, and PA, which highlight the importance of considering the unique cultural and historical backgrounds across different racial/ethnic groups when examining cognitive functioning in elderly.

## 1. Introduction

Subjective memory impairment (SMI), which is often referred to as subjective memory complaints or perceived cognitive impairment, is a self-reported cognitive problem characterized by complaints associated with declines in memory, attention, language, and problem-solving skills [1,2,3]. Individuals with SMI experience frequent forgetfulness, losing a train of thought, and confusion when making decisions [1]. SMI has been positively associated with the development of mild cognitive impairment (MCI) and Alzheimer-related disease [4,5,6]. For example, a 7-year follow-up study with healthy subjects found that people with SMI developed MCI or dementia significantly more rapidly than those with no SMI, with an observed average time to MCI or dementia being 3.5 years shorter for people with SMI [7]. Although there are inconsistent findings on whether SMI is the precedent of future MCI or Alzheimer-related disease [8], it appears that for certain groups, including older adults (e.g., aged 75 or older) or individuals with low education [9,10], SMI plays a crucial role in future cognitive decline and/or dementia. Thus, identifying risk factors of SMI and developing intervention programs targeting SMI risk factors are important first steps to reducing the onset of MCI and dementia.

The prevalence and incidence of cognitive impairment, including dementia and Alzheimer’s disease, have been reported with variations across different ethnic groups, indicating racial and ethnic disparities. For example, African American elders tend to show lower cognitive performance and are at higher risk for Alzheimer’s disease compared to Caucasians [11,12,13]. A mixed-race study investigating ethno-racial differences in cognitive functioning and rates of cognitive decline found that Hispanic and non-Hispanic Black individuals had lower levels of cognitive functioning compared with their age-matched White counterparts [14].

With substantial evidence for ethnic differences in cognitive health in late life, efforts to understand factors that contribute to these disparities have focused primarily on the role of differences in psychosocial factors (e.g., disadvantaged socioeconomic status and stressful life events) [15,16], education [17], hypertension risk [18], as well as genetic and biological differences [19,20,21] across ethnic groups. Specific causes of observed ethnic disparities in cognitive function remain unclear, and the impact of modifiable factors is underexplored. In addition, compared to the research on cognitive function and Alzheimer’s disease, only a few studies have examined ethnic differences in SMI and factors contributing to these differences.

To date, only one study has examined national trends in SMI, reporting greater rates of SMI in ethnic and racial minorities, including Hispanic, Black, and Asian groups, compared to non-Hispanic White Americans over the period from 1997 to 2015 [22]. Regarding contributing factors, several studies have suggested that emotional and lifestyle factors are associated with SMI. For example, a study by Chen and colleagues found that depression, lower educational level, low physical activity, and hypertension were significantly associated with SMI [23]. Yates and colleagues found that individuals with SMI reported anxiety or depression at higher rates in comparison to those without SMI. Individuals who reported both anxiety and depression at baseline were more likely to have SMI two years later [24]. Another study found that over a two-year follow-up period, individuals with SMI had two times a greater risk of developing depression compared to those without SMI [25]. Poor sleep quality also has been associated with SMI [13,14,15,16,26,27,28,29,30], as well as with worsening of memory [31] and an observed increase in amyloid-beta (Aβ) deposition in the brain, a feature of Alzheimer’s disease [32,33]. Although the literature suggests that physical activity is associated with decreased MCI and Alzheimer’s disease [34,35,36,37,38,39,40,41], no study has examined the association between physical activity and SMI. The positive impact of physical activity on cognitive decline and dementia may be attributed to stimulation of cerebral blood flow, neurogenesis, synaptic plasticity [36] and lowered risk of depression [42,43,44]. These findings raise the possibility for an association between physical activity and SMI as well.

According to these studies, potential explanations for ethnic differences in SMI may involve depressive symptoms and lifestyle behaviors. In fact, despite the high rates of symptoms of depression and anxiety [45,46,47], ethnic minorities, including Asian and African Americans, are less likely to seek treatment for psychological disorders [48,49,50,51]. Untreated depression, however, can interfere with cognitive health, contributing to SMI and cognitive decline. Studies further suggest that racial disparities in sleep and physical activity are associated with SMI. African Americans are more likely to have lower sleep quality and sleep variances than whites [31,52,53,54,55,56] and poor sleep quality is associated with increased risk of SMI in elderly African Americans [57]. African Americans also are less likely to be physically active compared to whites [58,59,60], partially due to lack of facilities that promote physical activity in their neighborhoods [59,61]. The high rates of depressive symptoms, sleep disturbance, and physical inactivity prominent in specific ethnic groups may contribute to different levels of SMI in different ethnic groups.

However, the hypotheses regarding ethnic differences in SMI and relevant factors has not been explored, particularly within ethnic minority groups. Previous studies have mainly focused on the comparison between hon-Hispanic whites and other minority groups. Further, Asians often are aggregated as one group, despite the unique cultural and historical backgrounds of each subethnic group and their potential impact on cognitive functioning. Therefore, in the present study, we investigated ethnic differences in SMI and contributing factors in African American, East Asian (Chinese, Korean) and Vietnamese American older adults. Specifically, we examined: (1) whether levels of SMI are different across ethnic groups; (2) whether depressive symptoms, sleep disturbance, and physical inactivity are more prominent in one specific ethnic group compared to other ethnic groups; (3) whether SMI is associated with depressive symptoms, sleep, and physical activity across ethnic groups; and (4) whether the impact of depressive symptoms, sleep, and physical activity on SMI differs across ethnic groups. This study is exploratory given that there is not enough evidence available in the literature on this topic that allow us to generate directionally specific hypotheses. Clarifying ethno-racial differences in SMI and addressing potential risk factors for SMI specific to Asian American subgroups and African Americans helps lay the foundation for the development of tailored interventions to reduce cognitive impairment and to improve quality of life in each ethnic group.

## 2. Methods

### 2.1. Participants and Recruitment

Participants were recruited through community-based organizations (CBOs), including senior centers, local ethnic organizations, and churches. Based on the long-term collaboration history, CBOs significantly contributed to recruitment and data collection. Specifically, CBO leaders were involved in the recruitment process by coordinating events or meetings for the research staff to visit and provide information about the study to potential participants. During the events or meetings, research staff offered detailed information about the study, conducted informed consent, and administered questionnaires. A total of one hundred fifty Asian Americans and 93 African Americans participated in the study. Inclusion criteria included: (1) age 50 or older, (2) residing in the greater Philadelphia region and (3) self-identifying as Asian American or African American. The total study sample of Asian Americans included East Asian [(Chinese (*n* = 43), Korean (*n* = 42)], and Vietnamese (*n* = 65) Americans.

### 2.2. Measures

In addition to the English version, all questionnaires were translated into Korean, Mandarin, and Vietnamese languages for the participants who preferred to use their heritage language.

Demographic information included age, ethnicity, gender, marital status, education level, and family income. Marital status was measured by asking if the participant was single, married, or divorced/separated/widowed and was coded into two categories: currently single and married. Education level also was coded into two categories: high school or under, and college or above. Ethnic group was categorized as East Asian (Chinese + Korean), Vietnamese, and African American. We decided to examine Asian subgroups due to similarities in immigration and socioeconomic characteristics among East Asian and Vietnamese Americans. Chinese and Koreans share similar cultural background and have a history of migrating voluntarily for economic or educational advancement, whereas, Vietnamese have a history of migration as refugees who experienced political hardships and war [62].

Subjective Memory Impairment (SMI) was assessed with the Everyday Memory Questionnaire 13-item version (EMQ-R) [63]. The EMQ-R is a self-report measure of memory failures in everyday life. Each item is rated on a Likert type response scale, which provides a score of 0–4, ranging from “never happened” to “happened very often” during the past month. The items were summed and given a score in the range of 0–52, with high scores indicating higher SMI. In addition to the total score, percent of participants who reported any symptoms of SMI was calculated.

Depressive Symptoms were assessed using the Patient Health Questionnaire (PHQ-9) [64]. The PHQ-9 is a self-report measure of the severity of depression. Each item measures the frequency of depressive symptoms over the last two weeks, and the answers range from “not at all (0)” to “nearly every day (4).” A total score ranging from 0 to 27 was given.

Health-behaviors focused on sleep and physical activity. For sleeping behavior, the average hours of sleep and sleep troubles were assessed. Sleep duration was measured by asking how many hours of sleep the participant had on average during the past month. Trouble sleeping was measured by asking participants if they experienced “trouble falling or staying asleep” during the past two weeks. The answers were coded as “yes” or “no.” Physical activity was measured by asking “Which of the following best describes your activity level during the past 3 months?”, to which respondents could give one of five different answers. These consisted of: (1) mostly sitting, usually doing things in a seated position, only doing activities of daily living (e.g., grooming, dressing); (2) light physical activity, doing light housework (e.g., preparing food, dusting) or light gardening, going for a walk 2–3 times a week; (3) moderate physical activity about 3 h a week, doing common housework (e.g., vacuum-cleaning/sweeping floors, lawn mowing), going for long walks (at least 2 km), or cycling; (4) moderate physical activity 4 or more hours a week or heavier physical activity 1–2 h a week, including heavy gardening/housework/home maintenance or physical exercise involving some breathlessness and sweating; or (5) active sports several times a week, which makes you sweat heavily and become breathless during the exercise, at least 3 h a week.

### 2.3. Data Analysis

The frequency and mean of relevant variables were examined by ethnic groups using descriptive statistics. For continuous variables, a Shapiro-Wilk test was conducted to determine whether the variables were normally distributed. The results indicated that the main variables were normally distributed. To examine group differences, Chi-square test analyses were conducted for categorical variables, including demographic information, and ANOVAs were conducted for continuous variables, including the total EMQ-R scores. To examine ethnic group differences in SMI among three groups controlling for demographic variables, multinomial regression analysis (African Americans vs. Vietnamese Americans, East Asian vs. Vietnamese Americans, East Asian vs. African Americans) was conducted. Then, multiple regression analysis was conducted for each ethnic group to examine association of SMI with depressive symptoms, sleep, and physical activities.

Furthermore, to examine whether there were significant interactions between ethnicity and the main independent variables (e.g., depressive symptoms, sleep, and physical activity) controlling for demographic characteristics, an ANCOVA was conducted with age, gender, marital status, education, and income as covariates. For the interaction analysis, depressive symptom scores were categorized into minimal (0–4), mild (5–9), and moderate/severe (10 or higher), and sleep hours were divided into two categories (less than 6 h and 6 or more hours). The literature suggests that a sleep duration of 6 h or less is associated with an increased incidence of dementia later in life [65]. SPSS version 25 was used to conduct all analyses.

## 3. Results

### 3.1. Demographic Characteristics of Participants

Table 1 shows the demographic characteristics of total participants and each ethnic group. The mean age for the total participants was 67.6 years old. The African American cohort was the oldest (68.4 years), and the East Asian cohort was the youngest (66.5 years). Overall, women represented a majority of the overall sample at 63.7%, while men represented 36.3% of the sample. In the African American group, women represented 71.6% of the sample and men only 28.4%. In terms of marital status, slightly more than 50% of participants were married (52.5%); among African American participants, a majority reported being currently single (84.8%). Within the Asian American subgroups, East Asians were most likely to be married (90.5%), followed by Vietnamese Americans (67.7%). African Americans were significantly less likely to be married than Vietnamese and East Asian Americans (χ^2^ (3, *n* = 242) = 85.32, *p* < 0.001). Data on education demonstrated that overall, 61.4% of the participants had a high school education or less, and most of the sample made less than $40,000 a year.

### 3.2. Ethnic Differences in SMI

**Figure 1:**
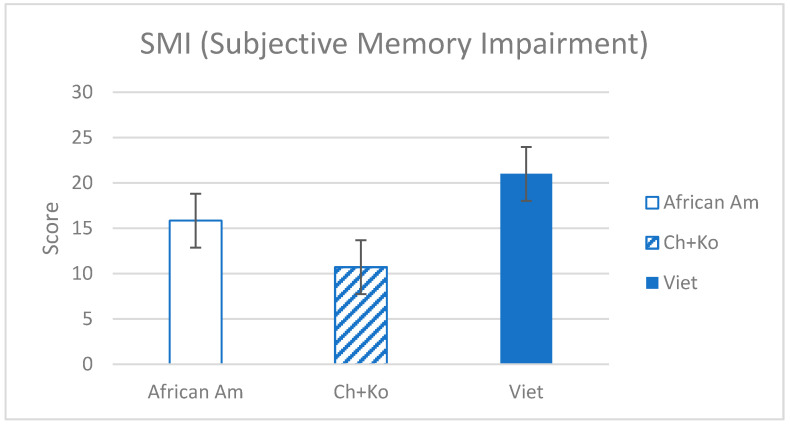
Subjective Memory Impairment by Ethnic Groups.

As shown in Table 1 and Figure 1, ANOVA analysis revealed that the SMI score was highest in Vietnamese Americans (M = 21.0) and higher in African Americans (M = 15.84) than in East Asian Americans (F(3,227) = 10.80, *p* < 001). In addition to the total score, there were significant differences in SMI symptom presence between African (21.9%), Chinese (48.8%), Korean (50.0%), and Vietnamese (91.1%) American groups (χ^2^ (3, *n* = 214) = 31.44, *p* < 0.001). To confirm ethnic differences in SMI controlling for demographic variables, multinomial regression analysis was conducted with dependent variables being African American (vs. East Asian), Vietnamese Americans (vs. African American), and East Asian (vs. Vietnamese Americans). Controlling for age, gender, marital status, education, and family income revealed significantly higher SMI score among Vietnamese Americans compared to African (AOR = 1.05, *p* < 0.05) and East Asian (AOR = 1.10, *p* < 0.001) Americans. African Americans reported significantly higher SMI scores than East Asian Americans (AOR = 1.06, *p* < 0.05) when controlling the demographic variables.

### 3.3. Sleep, Physical Activity, and Depressive Symptoms by Ethnic Groups

Vietnamese Americans (81%) were significantly more likely to face sleep problems compared to Chinese (32.6%), Korean (52.5%), and African (40.7%) Americans (χ^2^ (3, *n* = 237) = 32.36, *p* < 0.001). In reference to sleep hours, Vietnamese (M = 5.86) and African Americans (M = 4.99) slept significantly fewer hours than Chinese (M = 6.82) and Korean (M = 6.85) Americans. For physical activity, African (36.1%), Chinese (43.2%), and Korean (31.7%) Americans engaged in mostly light activity using housework. There was no significant difference in physical activity among the groups. Depressive symptoms were measured using the PHQ-9 questionnaire. Mean depression scores were 6.29 among Vietnamese Americans, 4.99 in African Americans, 4.84 in Korean Americans, and 3.39 in Chinese Americans. The differences in depression scores among ethnic groups were not statistically significant.

### 3.4. Association of SMI with Depressive Symptoms, Sleep, and Physical Activity by Ethnic Groups

The associations of sociodemographic factors, sleep, physical activity, and depressive symptoms with SMI for the total sample and each ethnic group were examined by regression analysis (Table 2). Overall, age (β = 0.895, *p* < 0.001), higher depressive symptoms (β = 0.417, *p* < 0.001), fewer average hours of sleep (β = −0.327, *p* < 0.05), and greater sleep trouble (β = 0.395, *p* < 0.05) were significantly associated with a higher level of SMI. For individual ethnic groups, among the demographic variables, age was significantly associated with SMI in African (β = 0.862, *p* < 0.05) and Vietnamese Americans (β = 0.508, *p* < 0.05). In the East Asian group (including Chinese and Korean), family income had a marginally significant association with SMI. Among the variables of main interest, average hours of sleep were associated with SMI in African Americans (β = −0.243, *p* < 0.05). Sleep trouble was significantly associated with SMI in African (β = −0.272, *p* < 0.05) and Vietnamese Americans (β = −0.498, *p* < 0.01). Physical activity was approaching significance with SMI in Vietnamese Americans. In all three groups, depressive symptoms were associated with SMI (African Americans, β = 0.368, *p* < 0.01; East Asian, β = 0.340, *p* < 0.05; Vietnamese Americans, β = 0.827, *p* < 0.001).

### 3.5. Interaction between Ethnicity and Independent Variables

**Figure 2:**
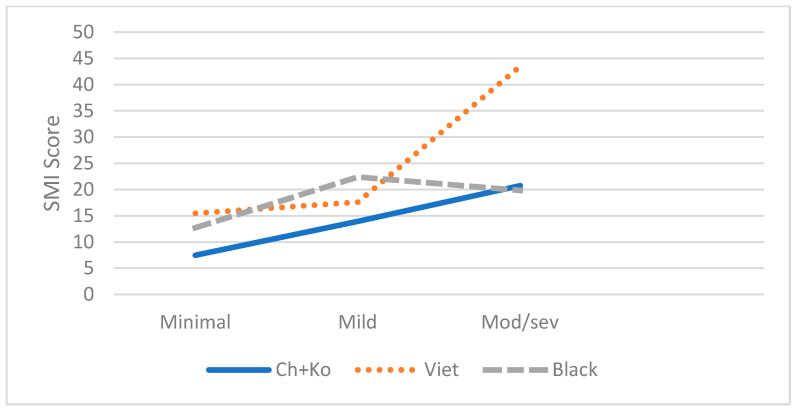
Effect of Depressive Symptoms on SMI by Groups.

**Figure 3:**
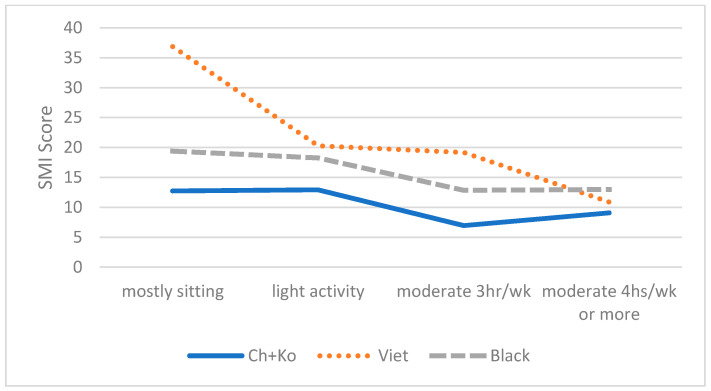
Effect of Physical Activity on SMI by Groups.

As shown in Table 3, controlling for demographic variables, the ANCOVA analysis revealed a significant interaction effect between ethnicity and depressive symptoms (F(4,204) = 7.81, *p* < 0.001, η2 = 0.15) and between ethnicity and physical activity (F(6,201) = 3.29, *p* < 0.01, η2 = 0.11) on SMI. As shown in Figure 2 and Figure 3, the links between SMI and depressive symptoms and physical activity were stronger for Vietnamese Americans compared to the other two groups. However, interactions between ethnicity and sleep hours or sleep trouble were not significant.

## 4. Discussion

This study aimed to contribute to the existing literature regarding subjective memory functioning and factors association with subjective memory functioning among racial and ethnic minority groups in the United States. Our study examined SMI and associated factors across different ethnic groups in the greater Philadelphia area. SMI and risk factors, including depressive symptoms, sleep, and physical activity, were assessed to examine their impact among older African and Asian American subgroups. There are several important findings from this study. Importantly, we found that older Vietnamese adults experienced the highest level of SMI, followed by African Americans. These findings are consistent with previous studies that have demonstrated significant ethnic differences in SMI [22] and subjective memory functioning [66,67]. However, while a previous study reported a higher level of SMI in African Americans compared to Asian Americans [22], our findings revealed that when Asian Americans were examined by subgroups, SMI level was higher in Vietnamese Americans than in African Americans.

While research to explain ethnic disparities in SMI is limited, previous investigation of the impact of stressful events and immigration-related stress on subjective memory function among Vietnamese Americans can illuminate our understanding of ethnic disparities in SMI. Research has shown that stressful life events, such as traumatic exposure, are linked to poor cognitive function and performance later in life [16,68,69] and accelerated cellular aging [70]. Research also suggested that the impact of stressful life events could be notably high among some populations, including those with lower socioeconomic status, leading them to experience clinical levels of cognitive impairment earlier in life [16]. Traditionally, Vietnamese Americans have a history of migration as refugees who experienced political hardships and war, whereas East Asians (Chinese and Koreans) mainly migrated voluntarily for economic or educational advancement [71]. Exposure to traumatic events associated with forced migration, family separation, and resettlement in Vietnamese communities may increase their vulnerability to cognitive impairment. High levels of depressive symptoms and sleep disturbance reported by older Vietnamese American adults in our study might be related to adverse life experiences and may serve as a prominent risk factor for SMI in this group.

It is also noteworthy that African Americans exhibited significantly higher levels of SMI compared to the combined East Asian Americans. This is consistent with previous studies that have demonstrated greater cognitive impairment in African Americans compared to European or Asian Americans [16,22]. In our study, African Americans reported higher levels of depressive symptoms and significantly fewer hours of sleep than East Asian Americans, and those risk factors were strongly associated with SMI in this group.

We further found that overall, participants in our study who endorsed greater depressive symptoms, fewer hours of sleep, trouble remaining asleep, and low levels of physical activity were more likely to report SMI when controlling for demographic characteristics. When the association of those variables with SMI was examined in each ethnic group, however, depressive symptoms were the only factor that remained significant across all ethnic groups, highlighting the influence of depression and emotional problems on SMI and MCI [24,72]. These findings are consistent with previous research studies that reported poor psychological health among older adults with memory complaints [73,74], and a study in which older adults with depression or anxiety were found to be nearly two times more likely to experience SMI than older adults without depression or anxiety [75]. In an attempt to explain this association, authors of a systematic review on SMI and affective symptoms [74] suggested a possible reciprocal relationship between SMI and depression. For example, depressive symptoms, including lack of motivation, energy, and concentration, may affect cognitive performance and consequently lead to an individual’s perception of cognitive problems [76]. The perception of a decline in memory, in turn, can precipitate concerns about dementia, having detrimental effects on depression and emotional health. Other researchers also have suggested that SMI disrupts daily functioning, which can be a source of emotional distress, and this distress can further aggravate perceived memory problems. Moreover, the perception of subjective memory functioning can contribute to withdrawal from positive health behaviors, including physical and social activity engagement [77,78,79], which can affect both depression and subjective memory functioning.

These studies and our findings indicate that, despite the undecided temporality of SMI and depressive symptoms, the presence or absence of depressive symptoms is the most significant variable differentiating older adults with or without SMI. Given that SMI [1,7,80] and depressive symptoms [81,82] are independent risk factors for MCI or Alzheimer’s disease and that depressive symptoms and SMI are more prevalent generally among racial and ethnic minority elders [17,65,83,84,85,86,87,88], the prevention and treatment of depression among racial and ethnic minority elders are critical for the prevention of cognitive impairment.

Another significant finding from our study was the interaction of ethnicity with SMI risk factors, particularly the impact of depressive symptoms and physical activity on SMI among Vietnamese Americans. The strength of these interactions suggests that Vietnamese American participants who are depressed and physically inactive are significantly more likely to experience SMI compared to certain other ethnic groups. In the current literature, it is unknown whether the impact of depression on subjective memory functioning is stronger or weaker in any specific ethnic group. However, based on recent research on the long-term trajectory of accumulated depressive symptoms on memory ability [89], it can be speculated that past immigration-related stress and accumulated emotional burden experienced by older Vietnamese Americans might not only increase their vulnerability to cognitive impairment but also amplify the negative impact of depressive symptoms on subjective memory function. These associations suggest that the major surge in SMI observed among Vietnamese participants who endorsed severe depressive symptoms and lower levels of physical activity and the endorsement of poor emotional and behavioral health among Vietnamese participants in our study may be related to traumatic stress experienced in the past during immigration. Thus, long-term, accumulated emotional distress potentially strengthens the negative impact of risk factors on subjective memory function in this group compared to the other ethnic groups.

Given that social and cultural factors accumulated during the life-course play an important role in cognitive aging [90], adverse events that Vietnamese and African American communities have experienced likely contribute to racial and ethnic differences in subjective memory functioning at older ages [91]. Specifically, systemic or structural racism and xenophobia may have played a major role in influencing these observed ethnic disparities. However, stressful life events, including traumatic experiences, were not directly assessed in our study. Thus, future studies are warranted to clarify the impact of adverse life events on cognitive health and subjective memory complaints in different ethnic groups. The present study has other limitations. This study focused on Chinese, Vietnamese, and Korean Americans who reside in the Greater Philadelphia Region, which may not be generalizable to those in other geographic areas in the U.S.. Although they represent three out of the five largest Asian ethnic groups, other Asian subgroups who have adverse life events, such as the refugee experience, were not examined in our study. Further, the study did not assess potential differences among sub-ethnic Black groups, which could also include individuals with refugee experiences. Furthermore, all measurements were collected through self-report without including neuropsychological assessments, which can introduce recall and social desirability bias. Future studies could utilize more objective measures for cognitive abilities, sleep, depression, and physical activity. The present study is also limited by a cross-sectional design, which makes it difficult to infer causal relationships between SMI and depressive symptoms or other health behaviors. Future longitudinal studies will help to establish the temporal relationship among SMI and the main contributing factors.

## 5. Conclusions

The present study contributes to the current literature by adding valuable information about subjective memory functioning in Asian subgroups and African Americans. Most previous studies on subjective memory function in different racial groups focused on the discrepancy between African Americans and white Americans. In addition, studies investigating ethnic-specific factors associated with cognitive impairment across different ethnic minority groups are extremely limited. With the projected increase in the number of older minority adults, the present study may serve as a guide for future studies in this field.

## Figures and Tables

**Table 1:** Participant Characteristics by Ethnic Groups.

	Total *n* = 243	African American (*n* = 93)	East Asian (Chinese + Korean) (*n* = 85)	Vietnamese (*n* = 65)	χ^2^, F
	*M* (*SD*), *n* (%)	*M* (*SD*), *n* (%)	*M* (*SD*), *n* (%)	*M* (*SD*), *n* (%)	
DEMOGRAPHICS					
Age	67.6 (9.40)	68.4 (9.67)	66.5 (9.70)	67.9 (8.59)	0.96
Gender					6.03 *
Male	86 (36.3%)	25 (28.4%)	30 (35.7%)	31 (47.7%)	
Female	151 (63.7%)	63 (71.6%)	54 (64.3%)	34 (52.3%)	
Marital Status					85.32 ***
Currently single	115 (47.5%)	78 (84.8%)	16 (18.8%)	21 (32.3%)	
Married	127 (52.5%)	14 (15.2%)	69 (81.2%)	44 (67.7%)	
Education					4.34
High school or under	145 (61.4%)	65 (71.4%)	42 (50.6%)	42 (67.7%)	
College or over	91 (38.6%)	26 (28.6%)	41 (49.4%)	20 (32.3%)	
Family Income					5.23
Less than $40,000	180 (81.8%)	66 (82.5%)	60 (75.0%)	54 (90.0%)	
$40,000 or higher	26 (18.2%)	8 (17.5%)	20 (25.0%)	6 (10.0%)	
Chronic Disease (yes)	145 (59.7%)	61 (65.6%)	36 (42.4%)	48 (73.8%)	
SMI					
Sx Presence (yes)	154 (72.0%)	57 (78.1%)	40 (50.6%)	57 (91.1%)	31.43 ***
Total Score	15.46 (11.65)	15.84 (10.81)	10.70 (9.83)	21.0 (12.49)	15.35 ***
Dep Total Scores	5.05 (5.96)	4.99 (6.01)	1.45 (0.72)	6.29 (6.57)	2.31
Depression Levels					5.20
Minimal (0–4)	140 (61.9%)	56 (62.9%)	51 (68.0%)	33 (53.2%)	
Mild (5–9)	47 (20.8%)	15 (16.9%)	14 (18.7%)	18 (29.0%)	
Moderate/severe (10+)	39 (17.3%)	18 (20.2%)	10 (13.3%)	11 (17.7%)	
Avg Hours of Sleep	6.42 (2.21)	4.99 (6.02)	6.84 (1.25)	5.86 (1.42)	8.14 ***
Sleep Duration					49.90 **
≤6 h sleep	57 (25.6%)	29 (36.7%)	10 (12.5%)	18 (28.1%)	
≥6 h sleep	166 (74.4%)	50 (63.3%)	70 (87.5%)	46 (71.9%)	
Sleep troubles (yes)	123 (51.9%)	37 (40.7%)	35 (42.2%)	51 (81.0%)	16.35 ***
Physical Activity Level					5.72
Mostly sedentary	36 (15.9%)	12 (14.5%)	13 (16.7%)	11 (16.9%)	
Light activity/housework	76 (33.6%)	30 (36.1%)	29 (37.2%)	17 (26.2%)	
Moderate act. for 3hrs/wk.	68 (30.1%)	21 (25.3%)	21 (26.9%)	26 (40.0%)	
Moderate act. for 4hrs/wk., active sports	46 (20.4%)	20 (24.1%)	15 (19.2%)	11 (16.9%)	

Note: * *p* < 0.05; ** *p* < 0.01; *** *p* < 0.001.

**Table 2:** Factors associated with SMI by ethnic groups.

Variables	Total	African American	East Asian	Vietnamese
	β	β	β	β
Ethnicity				
East Asian	−0.202			
Vietnamese	0.165			
Age	0.055	0.117	0.149	−0.017
Gender	0.061	0.017	0.028	0.08
Marital Status	0.047	−0.074	0.041	−0.012
Education	−0.081	−0.21	−0.145	−0.102
Family Income	0.065	−0.005	0.181	−0.022
Chronic Dis. (yes)	−0.065	−0.066	−0.001	−0.139
Sleep hours	−0.189 **	−0.329*	−0.220	−0.015
Trouble in sleep	−0.202 **	−0.316	0.074	−0.317 *
Physical Activity	−0.182 **	−0.091	−0.240	−0.248 *
Depressive Sx	0.503 ***	0.385 *	0.361 *	0.651 ***
Adjusted R^2^	0.44	0.16	0.23	0.6

Note: * *p* < 0.05; ** *p* < 0.01; *** *p* < 0.001.

**Table 3:** ANCOVA examining interaction effects of ethnicity and main independent variables on SMI.

Sources	SS	df	MS	F	η2
Ethnicity × Dep symptoms	2386.86	4	596.71	7.81 ***	0.15
error	13,291.51	204	76.39		
Ethnicity × Sleep hours	355.79	2	177.89	1.74	0.02
error	17,924.08	205	102.42		
Ethnicity × Sleep trouble	436.77	2	218.39	1.87	0.02
error	21,733.85	216	116.85		
Ethnicity × Physical activity	1882.83	6	313.68	3.29 **	0.11
error	16,287.83	201	95.81		

Note: (1) Age, gender, marital status, education, and income were used as covariates for all. Analyses; (2) ** *p* < 0.01; *** *p* < 0.001.

## Data Availability

The data presented in this study are available on request from the corresponding author.
